# Supplementary material for: Insight on the Interplay between Synthesis Conditions and Thermoelectric Properties of α-MgAgSb
Source: Materials (Basel). 2019 Jun 7;12(11):1857. doi: 10.3390/ma12111857 (PMC6600979; doi:10.3390/ma12111857)
Supplement: Supplementary file 1 [file materials-12-01857-s001.pdf]

materials

Insight on the Interplay between Synthesis Conditions and Thermoelectric Properties of α-MgAgSb

Julia Camut [1,2,*], Ignacio Rodriguez Barber [1,2], Hasbuna Kamila [1], Aidan Cowley [2], Reinhard Sottong [1], Eckhard Mueller [1,3] and Johannes de Boor [1,*]

[1] Institute of Materials Research, German Aerospace Center, Linder Hoehe, Cologne 51147, Germany; Ignacio.Barber@dlr.de (I.B.R); Hasbuna.Kamila@dlr.de (H.K); reinhard.sottong@web.de (R.S.)
[2] European Astronaut Centre, Linder Hoehe, Cologne 51147, Germany
[3] Institute of Inorganic and Analytical Chemistry, Justus Liebig University Gießen, Heinrich-Buff-Ring 17, Giessen 35392, Germany
* Correspondence: julia.camut@dlr.de (J.C.); johannes.deboor@dlr.de (J.d.B.); Tel.: +49–2203–601–4037 (J.d.B)

Received: 13 May 2019; Accepted: 3 June 2019; Published: date

Supplementary materials

X-ray diffraction (XRD) patterns

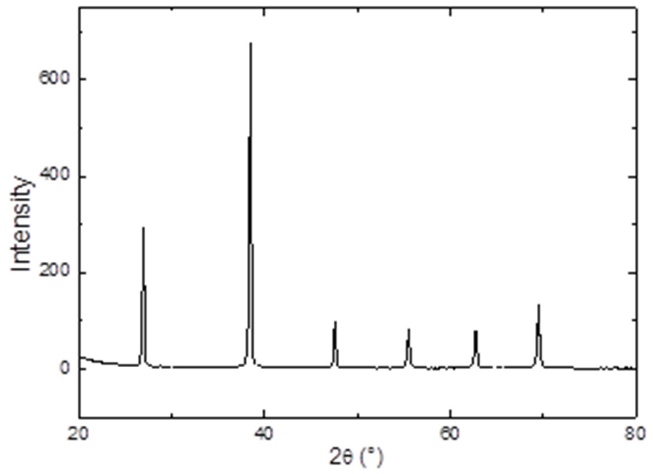

Figure S1. XRD pattern of the MgAg precursor synthesized with gas atomization**.**

In **Error! Reference source not found.**S1 is displayed the XRD pattern of the MgAg powder used for the synthesis of our materials. All diffraction peaks are pure MgAg. The powder contains less than 2 wt.%of pure antimony impurities, which is easily explained by the fact that antimony is used to clean the gas atomizer between runs.

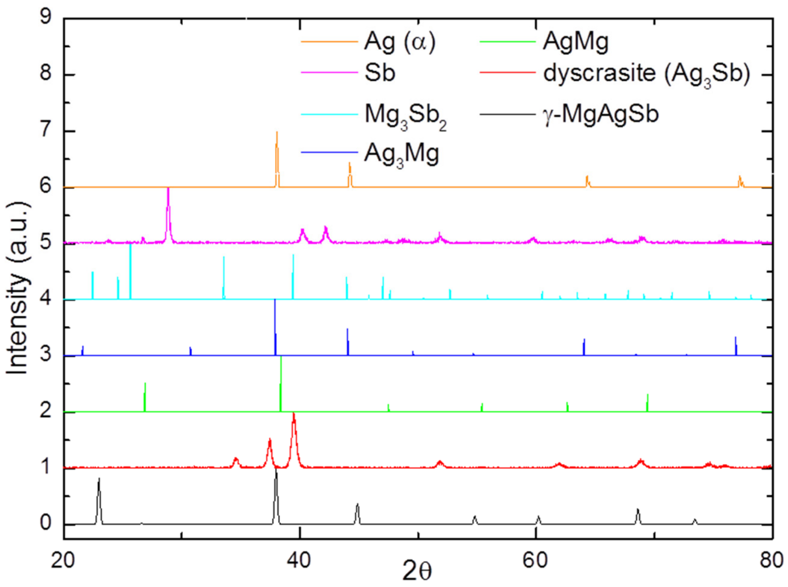

Figure S2. XRD patterns of common or plausible secondary phases in α-MgAgSb.

Error! Reference source not found.S2 shows the XRD patterns of common secondary phases in the Mg-Ag-Sb system. It is seen that a lot of secondary phases have very close main diffraction peaks. Pure silver and Ag_3Mg have similar patterns. They share their main peak with γ-MgAgSb and AgMg, which can however be distinguished by their lower intensity peaks. Dyscrasite, pure antimony and Mg_3Sb_2 are easily identifiable.

Thermoelectric properties for different dyscrasite contents

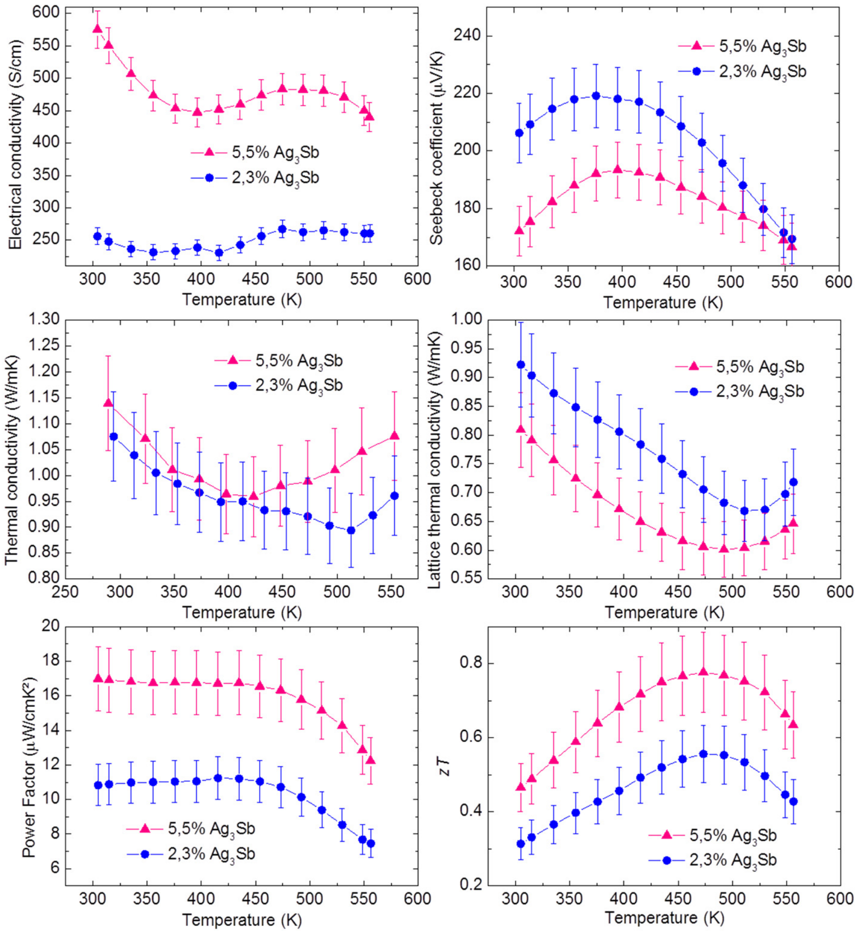

Figure 1 Comparison of thermoelectric properties for different dyscrasite contents. The samples were made with PBM.

Figure 1 displays the thermoelectric properties with different dyscrasite contents. It is seen that with increasing dyscrasite content, electrical conductivity increases and the Seebeck coefficient decreases (increase of carrier concentration). The lattice thermal conductivity decreases, although the overall thermal conductivity increases due to the electrical contribution. The Power Factor and zT are overall increases with increasing dyscrasite content.
